# A Rare Complication of Robot-Assisted Total Knee Replacement: Spontaneous Recurrent Hemarthrosis and Its Management

**DOI:** 10.7759/cureus.82933

**Published:** 2025-04-24

**Authors:** Muhammad Zain-ur-Rehman, Corinna Winkworth, Nadim Aslam, Rahul S Chivate

**Affiliations:** 1 Trauma and Orthopaedics, Worcestershire Acute Hospitals NHS Trust, Worcester, GBR; 2 Interventional Radiology, Worcestershire Acute Hospitals NHS Trust, Worcester, GBR

**Keywords:** angiography, geniculate artery, pseudoanuerysm, robotic total knee replacement, spontaneous recurrent hemarthroses

## Abstract

Spontaneous recurrent hemarthrosis following total knee replacement is a relatively rare complication. It requires a different and swift management plan compared to any other knee swelling. Presentation can vary from 2 months to 1.5 years, and it can lead to further complications, including a limited range of movement and severe stiffness, resulting in compromised functionality. Although etiology is not well understood, it can be due to instrumentation leading to direct vessel injury, including popliteal and geniculate arteries, arteriovenous fistula, and pseudoaneurysms. This case report aims to contribute to the early identification and management of this rare complication. We present a case involving the early onset of recurrent spontaneous hemarthrosis following robot-assisted total knee replacement surgery.

## Introduction

Spontaneous recurrent hemarthrosis following total knee replacement is a relatively rare complication, being reported in up to 1.6% of patients [1]. It requires a swift management plan compared to any other knee swelling. Presentation can vary from 2 months to 1.5 years [2]. It can lead to further complications, including limited range of movement and severe stiffness, resulting in compromised functionality [3,4]. Even though etiology is not well understood, it can be due to instrumentation leading to direct vessel injury, including popliteal and geniculate arteries, arteriovenous fistula, and pseudoaneurysms [5,6]. This case report contributes to the early identification and management of this rare complication. We present a case of early identification and management of recurrent spontaneous hemarthrosis following robot-assisted total knee replacement surgery.

This article was previously presented as a meeting abstract at the Annual Sicot Congress in Serbia in September 2024.

## Case presentation

**Figure 1:**
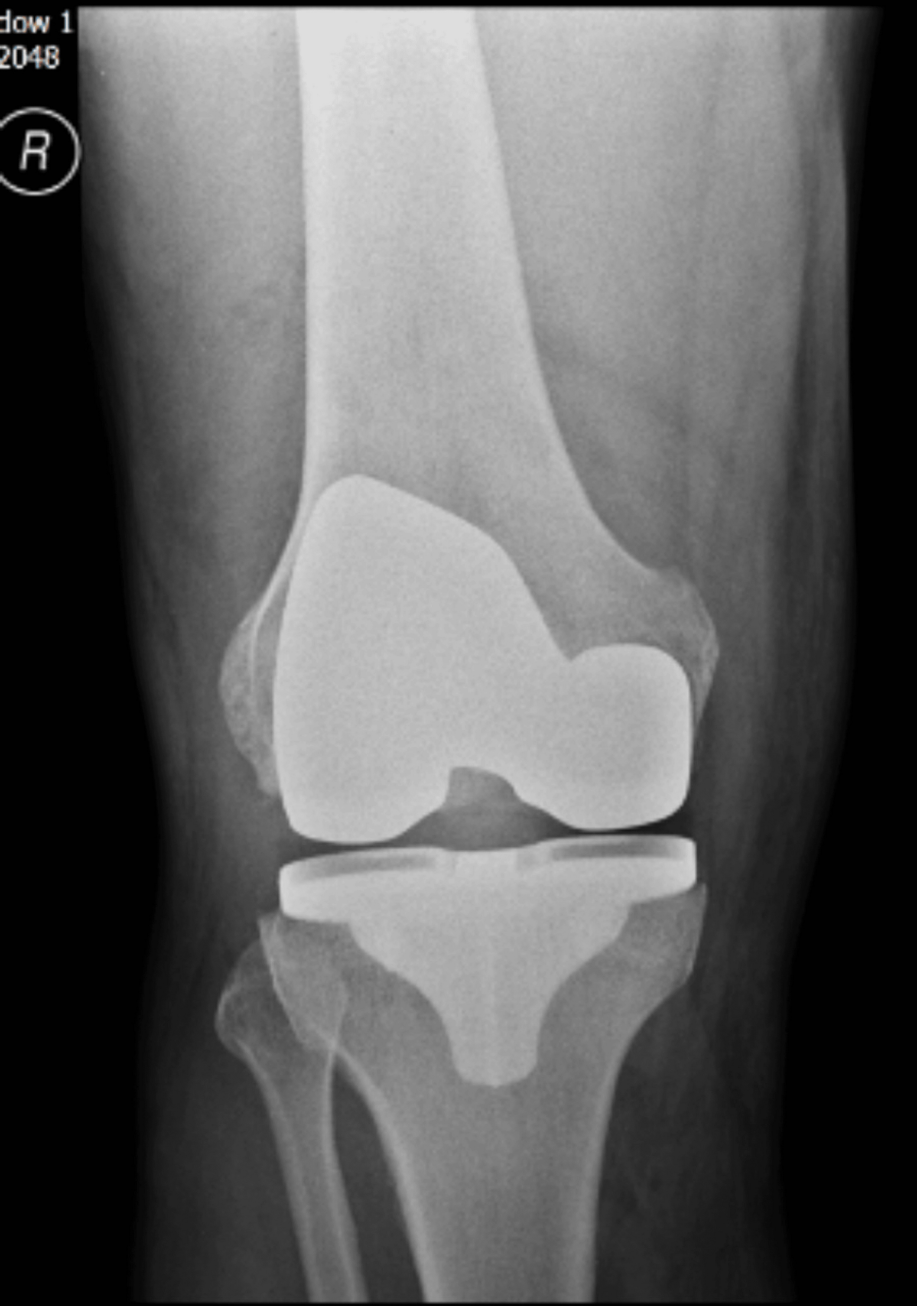
Postoperative anteroposterior radiograph of the knee showing total knee replacement in place and gross joint effusion in the form of loss of the supra and infrapatellar fat pad.

**Figure 2:**
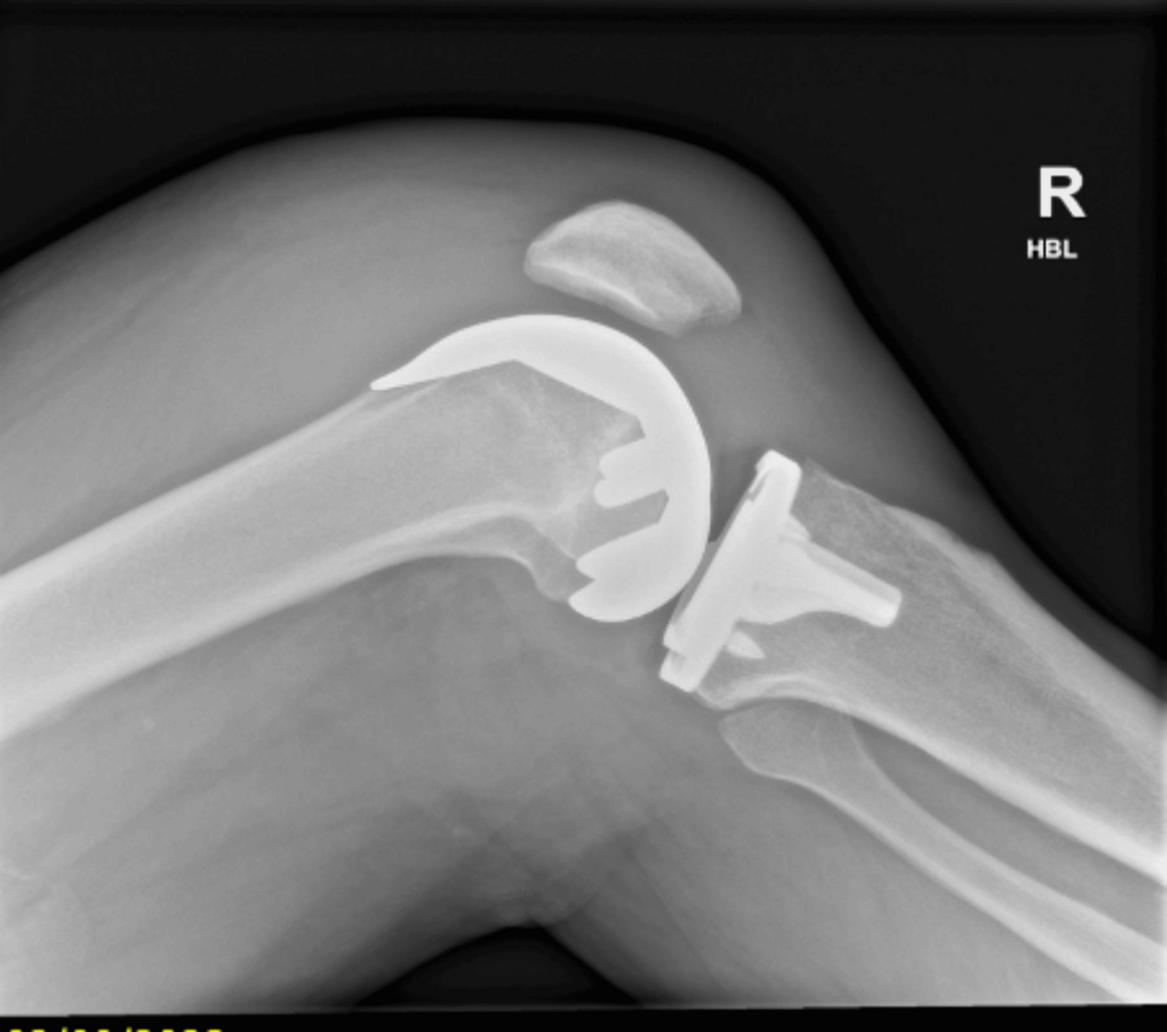
Postoperative lateral radiograph of the knee showing total knee replacement in place and gross joint effusion in form of loss of supra and infrapatellar fat pad.

A 69-year-old male underwent right-sided robotic-assisted total knee replacement for severe grade 4 knee osteoarthrosis in August 2023. His surgery was uneventful, and he was discharged home with routine physiotherapy protocol and venous thromboprophylaxis. He was recovering well until four weeks after the surgery, when he presented in the emergency department with the acute onset of pain and swelling of the right knee. He was able to bear weight and did not have any fever spikes. There was no history of bleeding disorder, and he had already completed his two weeks of venous thromboembolism prophylaxis medications. Clinical examination showed moderate-to-large diffuse swelling around the knee, mildly raised temperature compared to the other side, and no significant erythema or redness. The knee range of motion was 10-90 degrees. His blood tests for inflammatory markers showed a C-reactive protein level of 5 mg/dL and a white blood cell count of 6.8 × 10^9^/L. Radiographs of the right knee showed loss of the supra and infrapatellar fat pad, suggestive of gross joint effusion (Figures 1, 2). His knee was aspirated with around 100 mL of dark-colored frank blood, which raised the suspicion of venous bleeding. The cultures were negative. He was sent home with advice of icing and elevation of the knee at rest.

As he had recurrent knee swelling, after discussion with the vascular surgeons and interventional radiologists, they advised a lower limb CT angiogram to see any vascular abnormalities. It showed no large vascular abnormality. However, there was moderate hemarthrosis. The CT images were significantly obscured due to streak artefacts from metallic implants.

**Figure 3:**
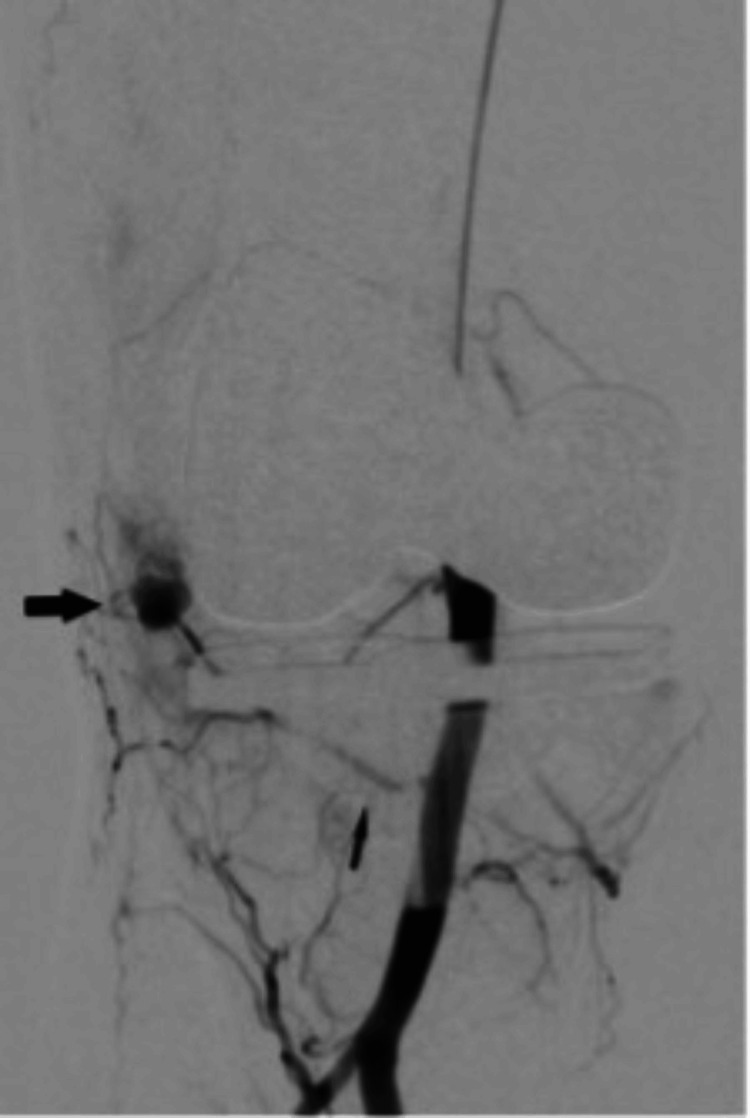
Angiography done through a catheter in the popliteal artery shows a pseudoanuerysm (solid arrow) arising from the inferior medial geniculate artery (thin arrow).

**Figure 4:**
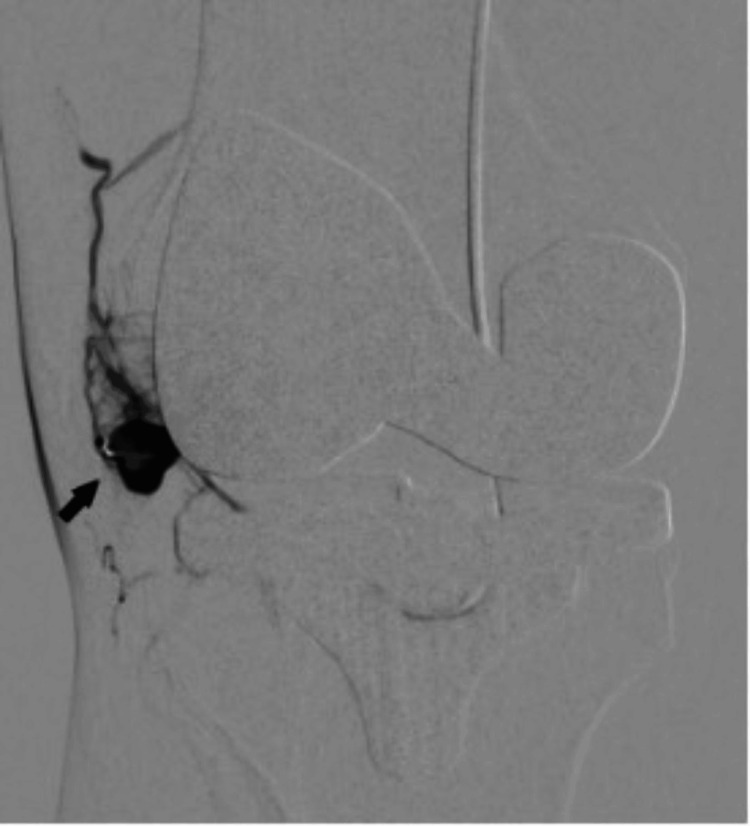
Superselective angiography through the inferior medial geniculate artery shows intense opacification of the pseudoaneurysm (solid arrow).

**Figure 5:**
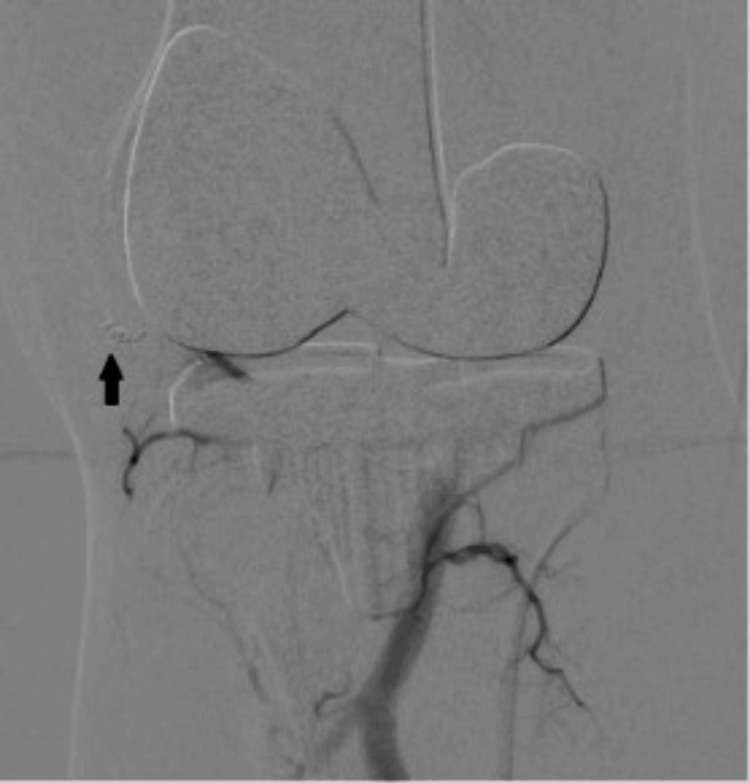
Post-embolization, using particles, embospheres, and coils (solid arrow), angiography shows non-opacification of the pseudoaneurysm.

Given continued knee joint swelling, the patient underwent right lower limb angiography the following week through the right femoral artery approach, which identified a pseudoaneurysm arising from the inferior medial genicular artery. This was embolized using 250 µ particles, embospheres, and 2 mm pushable coils. Selective angiogram performed from the superior medial and lateral genicular artery showed abnormal blush, and pruning was done using 250 µ embospheres (Figures 3-5). An ice pack was placed over the knee while instilling embolic agent particles to cause vasoconstriction of the skin arterial supply to avoid ischemia.

At the sixth-month follow-up appointment, he had fully recovered, was able to perform his daily activities, and did not have any further swelling episodes.

## Discussion

Recurrent spontaneous hemarthrosis can be difficult to manage if not diagnosed promptly, leading to severe limitation of movements and stiffness of the knee. After ruling out infection, a course of conservative treatment is advised; failure of which can lead to appropriate intervention [4]. Several approaches for recurrent spontaneous hemarthrosis of the knee after total knee replacement have been described in the literature, including Saksena et al. [1], who described an algorithmic approach to stop all anticoagulation medications and to test patients’ blood to rule out any coagulopathies. After icing and resting the knee, if a large hemarthrosis persists, it should be aspirated and immobilized.

Angiography by involving an interventional radiologist has been described as the first-line procedure in the diagnosis of such cases [7]. This technique is also useful in ruling out arteriovenous fistulas and pseudoaneurysms [8,9]. Once the diagnosis is confirmed, selective arterial embolization of the geniculate arteries is the procedure of choice, which decreases blood flow to the soft tissues and synovium around the total knee replacement. Weidner et al. [4] described a case series that reported that geniculate arterial embolization led to the resolution of hemarthrosis in 12 of 13 patients (92.3%). The one clinical failure likely represented a case of misdiagnosed periprosthetic joint infection. In their study, the average interval between arthroplasty and embolization was 47 months (range: 2-103 months), and the average time from onset of hemarthrosis to embolization was 4.1 months (range: 1-11 months).

In another series of five patients reported by Bagla et al. [3], who presented with spontaneous hemarthrosis after total knee replacement, selective arterial embolization was performed with spherical embolic particles (diameter range: 100-700 μm). Angiography demonstrated synovial hypervascularity with geniculate artery tumor blush appearance in all patients. The average time to resolution of effusion was 2.6 weeks, with no recurrence reported during follow-up (mean: 25.4 months; range: 16-48 months).

In previous literature, spontaneous recurrent hemarthrosis after total knee replacement has been reported as a late complication [4]. However, in our case, the patient presented quite early, i.e., within four weeks of surgery. Kindsfater and Scott [10] reported that the average interval between implantation of the prosthesis and the first bleed was 24.2 months. Of 30 knees, nine responded to conservative care alone. The remaining 21 knees continued to have recurrent bleeds requiring surgical intervention. They described the histologic findings, including focal synovial hyperplasia and significant hemosiderin deposition.

On the other hand, Guevara et al. [10] showed the significance of identifying other cases, including blood dyscrasias, as their presence led to repeat embolization and limited clinical success. In a series of eight cases by Dhondt et al. [11], angiography revealed hypertrophic vascular synovium in seven patients, with an additional false aneurysm in one patient.

After embracing the innovation of robotic-assisted total knee replacement globally, we should be mindful of spontaneous recurrent hemarthrosis in cases of recurrent swelling. Treatment modalities, including conservative management, as mentioned above, should be trialed. In recurrent spontaneous hemarthrosis, angiography leading to selective arterial embolization has shown promising results.

## Conclusions

Recurrent spontaneous haemarthrosis is a rare complication that can occur following robotic-assisted total knee replacement. A trial of conservative treatment should be instituted, and infection and blood coagulopathy should be ruled out. Angiography followed by selective arterial embolization of the geniculate arteries is the treatment in appropriately selected patients and has shown promising results.
